# Medical Education in the Provinces

**Published:** 1894-09-29

**Authors:** 


### Medical Education in the Provinces.

Most of the larger provincial towns of England are now provided with fully-equipped medical schools, and, in large well managed hospitals, afford adequate opportunities for the study of disease. The provincial schools, unlike the metropolitan, have governing bodies quite distinct from the governing bodies of the hospitals at which, by arrangement, the students receive clinical instruction. It is not possible to do more than enumerate these schools here; but it is only right that it should be said with regard to them as a whole that they are in a thriving condition. The teaching, particularly in science, is excellent, and as a rule the fees are moderate.

Of the ten provincial schools, those at Oxford and Cambridge occupy a special position in virtue of their connection with ancient universities. Both universities provide instruction in medicine; and, in so far as the science and theoretical classes are concerned, they have done this on no niggardly scale. The physiological and anatomical departments at Oxford hold their own with those of any medical school in the kingdom. Cambridge has been even more ambitious than the sister University, for she encourages students not only to take their classes, but their hospital practice also at Cambridge. Oxford, on the other hand, advises her men to give two years at least to clinical study at one of the London hospitals. This is wiser, as Addenbrooke's Hospital at Cambridge is hardly large enough to afford the practice that a medical student needs. Candidates for the Oxford and Cambridge degrees must have, for at least nine terms, or three years, resided in one of the colleges. The conditions of undergraduate life at these seats of learning are sufficiently well known.

The University of Durham has its local habitation, not at Durham, but at Newcastle-on-Tyne, where there is a thriving school of medicine. Clinical instruction is provided at the Newcastle Royal Infirmary, which has 280 beds. Residence at Newcastle for at least one year is required of candidates for the Durham degrees, and, by complying with this condition, students who have had the greater part of their training at other recognised schools become entitled to sit for the Durham M.B.

Victoria University is an examining body which has three incorporated colleges—Owens College, Manchester; University College, Liverpool; and Yorkshire College, Leeds.

Owens College has an admirably-equipped medical school, and the teaching in all branches of science relating to medicine is excellent. The buildings have recently been considerably enlarged, and the new laboratories and class-rooms will be formally opened in November by the Duke of Devonshire. The Manchester Infirmary affords abundant opportunities for clinical study. There are two residential halls at which Owens College students may reside.

Except London, there is perhaps no town in Britain which affords a larger amount of material for the study of disease than the great seaport of Liverpool; and it is well that in Liverpool University College a good school of medicine has been established to utilise this material. The infirmary attached to the school contains 300 beds, and lock, lying-in, and eye and ear hospitals, to the practice of which students are admitted, are in the immediate vicinity.

No one can visit Leeds without being impressed with the healthy public spirit of its citizens. Of this there are evidences on every hand. Among the institutions in which Leeds takes a just pride is its medical school. New medical school buildings have just been completed, and will be formally opened in a few days by the Duke and Duchess of York. These buildings occupy a commanding site near the infirmary, and in their arrangement and fitting up leave nothing to be desired. Students receive clinical instruction at the Leeds General Infirmary, with 471 beds, and at several special hospitals also.

Mason College, Birmingham, is one of the best known educational institutions in this country. It has a reputation for science teaching which is of the highest. The medical department of the college is in a thoroughly efficient condition, and the provision in laboratories, and otherwise for practical work, is ample. As the centre of a huge industrial population Birmingham is well provided with hospitals. These have been well utilised for clinical study; for it is claimed for the medical school that its students have the opportunity, if they choose, of seeing the practice afforded by institutions with a joint total of 1,560 beds, and considerably over 100,000 out-patients annually.

The School of Medicine at Sheffield occupies buildings that were opened in 1888, in which every provision for medical education has been made. Clinical instruction is obtained at the Sheffield General Infirmary, which has 200 beds, and at the Public Hospital and Dispensary, with 105 beds. Moreover, at the Jessop Hospital for Women, with 45 beds, there are opportunities for practical work in gynæcology and obstetrics.

Those who attended the recent annual meeting of the British Medical Association at Bristol were enabled to learn something of the excellent work in medical teaching that is done at University College, Bristol. This thriving school has an excellent record in connection with all the public examinations. There are two large hospitals in Bristol—the General Hospital, with 200 beds, and the Royal Infirmary, with 170.

The recently-established Medical School at Cardiff is affiliated with University College. It is the hope of the friends of the school that in due course it will become a fully-equipped medical school for South Wales. In the meantime instruction is provided in all the subjects of the first three years of study. There is an hospital close to the school, which will shortly have 200 beds.

---

Statistics of the Imperial German Health Department show Berlin as the healthiest city in the world. Here the death-rate is 16·3 per 1,000. Compared with Alexandria, a city fanned by the sea breeze, and constantly under the influence of sunshine, Berlin shows to no small advantage. In this latter city statistics give the death-rate at 52·9 per 1,000.

